# The gametogenic cycle and spawning of the short-necked clam, *Paphia undulata* Born, 1778 (Bivalvia: Veneridae) from Timsah Lake, Suez Canal, Egypt

**DOI:** 10.1186/s40850-023-00182-9

**Published:** 2023-09-13

**Authors:** Mostafa A. M. Mahmoud, Mohamed H. Yassien

**Affiliations:** National Institute of Oceanography and Fisheries, Cairo, Egypt

**Keywords:** Sexual maturity, Spawning cycle, Hermaphroditism, Suez Canal

## Abstract

**Background:**

*Paphia undulata*, The Short-Necked Clam, is an edible marine bivalve that is consumed internationally and locally in Egypt. Overfishing and pollution have caused population declines in Egyptian fisheries during the last decade. Accurate reproductive biology knowledge is critical for designing long-term exploitation strategy for this resource. *P. undulata* spawning and gametogenic cycle research were carried out from January to December 2020 along Timsah Lake, Suez Canal, Egypt.

**Results:**

These clams are functionally dioecious with a very low incidence of hermaphroditism. The sex ratio of the clam population was 1.0:1.07:0.04 for male, female and hermaphrodite respectively. The shell lengths of the collected clams were 4.64 ± 0.83 cm in males, 4.55 ± 0.9 cm in females and 4.19 ± 0.3 cm in hermaphrodite clams. The sizes at the onset of sexual maturity in both males and females were 2.1 cm and 2.5 cm respectively.

**Conclusions:**

Reproductive studies revealed that this species has a prolonged spawning season that is not restricted to a specific period.

## Introduction

The bivalve clam *Paphia undulata* Born, 1778 (Bivalvia: Veneridae) is economically and ecologically important as a food source and biomass and may affect on communities. This species forms a large proportion of the shellfish markets in many coastal countries all over the world. Furthermore, worldwide consumption of marine bivalves has increased with growing interest in the nutritional and health benefits of these products. The overall bivalve production in Egypt was estimated between 2,600 and 25,200 tones. Bivalves are a major source of protein for the community [1–3]. It's a good source of protein, containing approximately 68.77% crude protein on a "dry weight basis" [4–7], and it's high in proteins, particularly essential amino acids, which are considered an essential source of nutrients for many people, particularly in developing countries [8]. The clams are consumed locally in Egypt, and its population has declined across the Egyptian fisheries in the past decade. This is mainly attributed to overexploitation, pollution, and parasites [9–12]. Gametogenesis is the process in which gametocytes undergo cell division and differentiation to form haploid gametes [13]. The sex ratio is a fundamental indicator for reproduction success in dioecious species, in which, nearly equal numbers of males and females are produced, giving a balanced sex ratio, whereas the sex ratio can skew towards one sex in hermaphroditic species [14, 15].

The size at the commencement of sexual maturity is typically defined as the length of the shell at which the gonad transitions happen from its primitive virgin condition (undifferentiated or juvenile gonad) to the stage at which it is sexually differentiated. The study of size at first maturity is important as it establishes a minimum size limit to prevent fishing for immature clams without permission [16]. Additionally, it shows population shifts caused by overfishing, and other factors. Many species reach sexual maturity at a specific age that corresponds to a wide range of individual lengths because not all members of a species develop at the same size (length). The size at start of maturity is often regarded as the "average size at which 50% of a population is mature," according to Roa et al. and Kandeel [16, 17].

The awareness of the clams’ culture was initiated in the 1970s after it was noticed that the worldwide population hastily declined. Increasing coastal populations, improved harvesting efficiency and pollution were some of the reasons that were contributing to the decrease and maybe the destruction of the clams’ local stock from their geographical range [18]. Due to the commercial importance of *P. undulata* and its poly-culture potential, there is a need to re-assess its status considering that there are still no known management policies governing its exploitation. This study aims to assess the reproductive cycle, gametogenesis, and spawning season of *P. undulata*. The result is essential to predict the reconstitution of the clam natural populations and it is recommended to support large-scale artificial breeding. and the development of artificial breeding facility in this area.

## Materials and methods

### Study area and sample collection

**Fig. 1:**
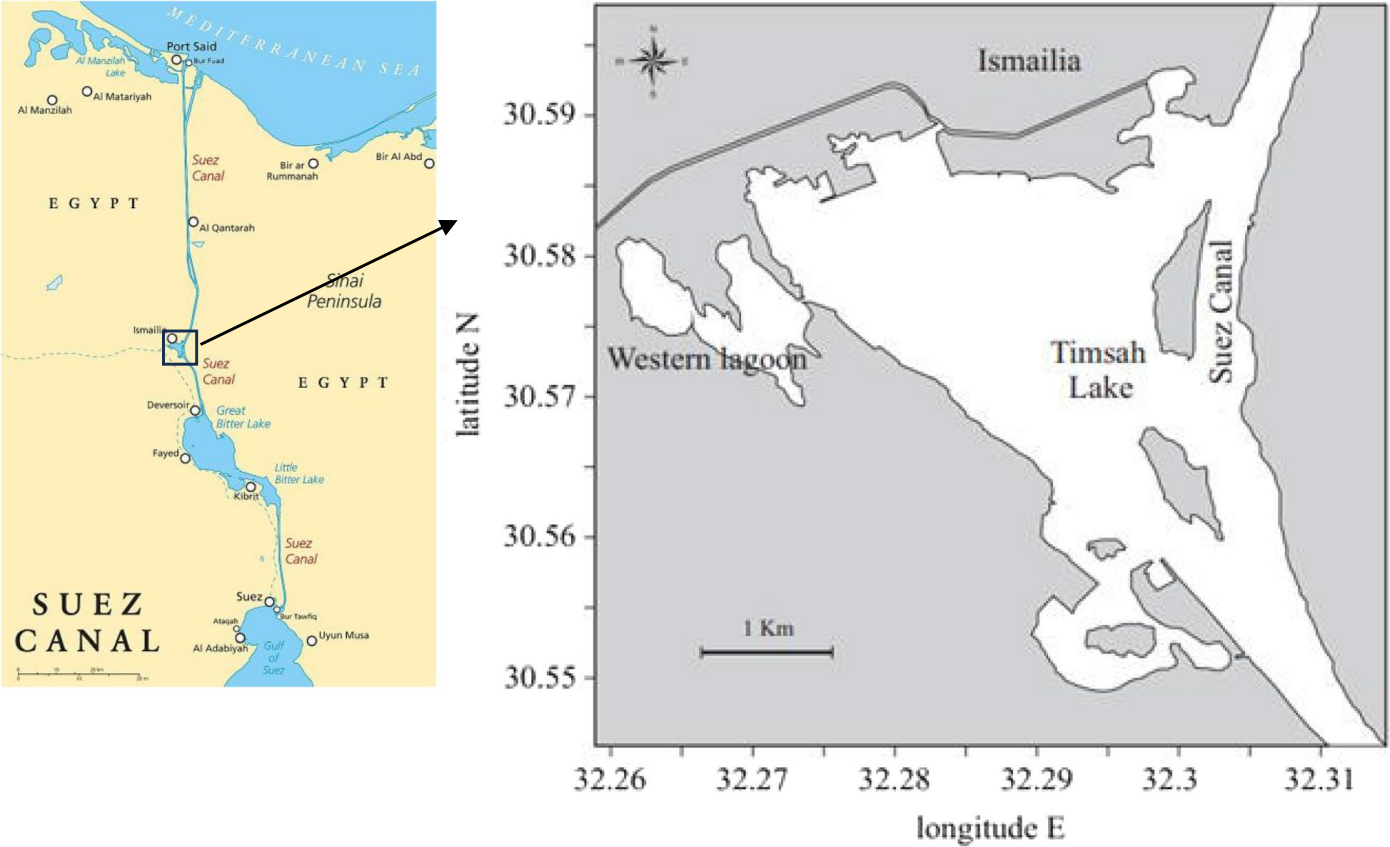
Map of the sampling site of *P. undulata* in Timsah Lake, Ismailia. (According to El-Sherbiny et al. [19])

Two hundred and eighty-four random samples of the clams were collected by shellfish local fishermen from Timsah Lake sandy shore at Ismailia City, Egypt (Fig. 1). During the period from January to December 2020 (lat. 32° 17` E; long, 30° 35`·N).

Timsah Lake is a small and shallow lake located near Ismailia City at the midpoint of the Suez Canal approximately 80 km south of Port Said City. It lies between 30º 33ʹE, 30º 35ʹ N latitude and 30º 16ˋand 30º 19ˋE longitude. Its surface area is approximately 16 km^2^ with depth variation between 3 and 16 m. from the western side, it is attached to a small shallow western lagoon which is nearly brackish. The significant-high variation of salinity in Timsah Lake from 40 ‰ in summer to 7.3 ‰ in winter was due to the presence of many freshwater sources such as Ismailia freshwater canal and wastewater discharge from the western lagoon [20].

The clams were transferred alive to the hydro lab of the National Institute of Oceanography and Fisheries in Hurghada and prepared for morphometric analysis. The shell length was measured to the nearest 0.05 mm using a vernier calliper. The total shell weight in grams was recorded using an electronic balance accurate to two decimal places. The soft tissues were removed from the shell to obtain the gonad-visceral mass, the gonad and visceral mass are not easily separated. The gonad mass was used to distinguish the sex via smear preparations. The gonads were prepared for histological analysis.

### Sex ratio and size at the onset of sexual maturity

Sexes can’t be distinguished by macroscopic examination; therefore microscopic histological examination of gonads was performed to determine sex. Chi-square (χ2) analysis was used to test for significant differences between monthly sex ratio of the studied species. The size at the onset of sexual maturity is basically described as the lowest size or age at which fifty percent of individuals matured sexually. The data were plotted against shell length and then assessed by fitting a logistic curve to the percentage of mature by size using the methods discussed by Jagadis et al. and Torroglosa and Giménez [21, 22].

### Histological studies

Standard procedures for histological preparation of *P. undulata* gonad tissues were followed [15, 23]. The protocol included: fixing 1 cm pieces of tissue in Bouin’s fluid for 24 h, dehydrating them in a graded series of alcohol, clearing in xylene, and embedding them in paraffin wax. Sections of 5 μm thickness were stained with hematoxylin–eosin. The histological slides were observed and photographed using a mounted digital camera in a light microscope (Model Optica B-150DB) with magnification from X4 to X40.

Gonadal development of *P. undulata* has been described and classified into five stages: (1) early active stage (EAS); (2) late active stage (LAS);(3) ripe (maturing) stage (RS); (4) partially spent (spawning) stage (PSS); and (5) spent stage (SS), with some modifications, based on the criteria of *P. undulata* [24, 25]. The reproductive cycles of the sexes were analyzed independently because gonadal development times differed between the sexes.

## Results

### Sex ratio and size at the onset of sexual maturity

Microscopic examination of *P. undulata* revealed no color differentiation, and both male and female gonads were creamy in colour regardless of sex, stage, and size. Thus, it is neither possible to determine the sex and nor characterize the gonad development of the present species microscopically. These clams were functionally dioecious with very low incidences of hermaphroditism. However, this species does not exhibit sexual dimorphism. The ripe gonads occupied almost the whole tissue of the mantle.

**Fig. 2:**
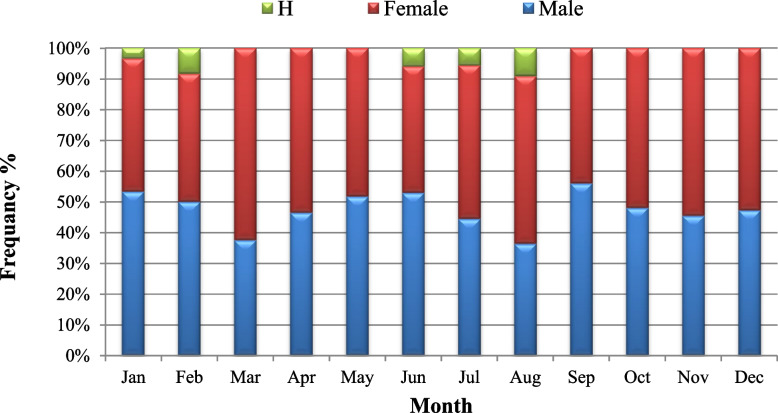
Monthly frequency of *Paphia undulata* males, females and hermaphrodite (H) sampled from January to December 2020 from Timsah Lake

**Fig. 3:**
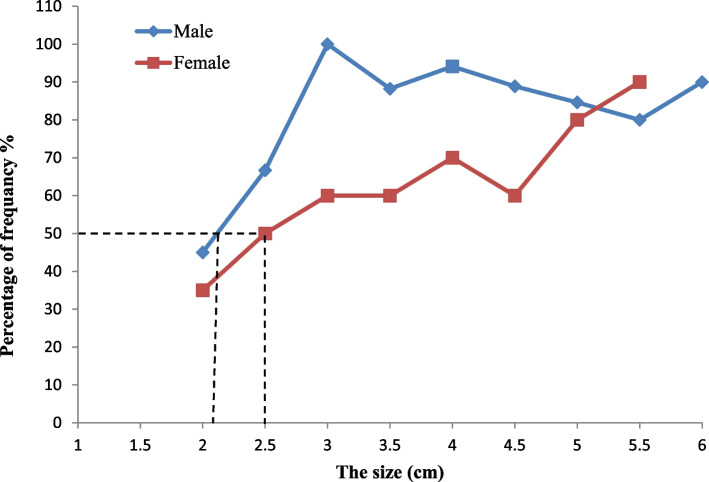
The size at the onset of sexual maturity of *Paphia undulata* from Timsah Lake

The results of 285 examined individuals of these clams showed that 135 (47.4%) clams were males, 144 (50.5%) were females, and only six individuals were hermaphrodites thus representing (2.1%) of the studied sample (Fig. 2). Hermaphrodite specimens were collected during January and February in the winter season and from June to August in the summer season. The sex ratio (male: female: hermaphrodite) of the clam population was 1.0:1.07:0.04 (*p* = 0.269) (Table 1). Shell lengths of the collected clams ranged from 2.31 to 6.22 mm (4.64 ± 0.83) in males, 2.05 to 5.97 (4.55 ± 0.9) in females and 3.70 to 4.36 (4.19 ± 0.3) in hermaphrodite clams. The average size at the onset of sexual maturity in *P. undulata* was 2.1 cm in males and 2.5 cm in females (Fig. 3).

**Table 1:** Monthly distribution of the different maturity stages and sex ratio for males, females and hermaphrodite (H) of *Paphia undulata* from January to December 2020

Months	Male					Total No	Female					Total No	H	Sex ratio M:F:H
	**EAS**	**LAC**	**RS**	**PSS**	**SS**		**EAS**	**LAC**	**RS**	**PSS**	**SS**			
**Jan**	**6**	**3**	**7**			**16**	**3**	**7**	**3**			**13**	**1**	**1:0.81:0.08**
**Feb**	**1**	**3**	**2**			**6**	**1**	**1**	**3**			**5**	**1**	**1:0.83:0.20**
**Mar**		**3**	**5**	**4**		**12**	**1**	**8**	**6**	**5**		**20**		**1:1.67:0.00**
**Apr**		**4**	**6**	**3**		**13**		**4**	**9**	**1**	**1**	**15**		**1:1.15:0.00**
**May**		**5**	**5**	**3**	**2**	**15**		**4**	**5**	**3**	**2**	**14**		**1:0.93:0.00**
**Jun**		**3**	**5**		**1**	**9**		**1**	**3**	**2**	**1**	**7**	**1**	**1:0.78:0.14**
**Jul**		**2**	**3**	**2**	**1**	**8**		**2**	**5**	**1**	**1**	**9**	**1**	**1:1.13:0.11**
**Aug**		**1**	**6**		**1**	**8**		**5**	**4**	**1**	**2**	**12**	**2**	**1:1.50:0.17**
**Sep**		**3**	**5**	**5**	**1**	**14**		**2**	**3**	**5**	**1**	**11**		**1:0.79:0.00**
**Oct**			**2**	**8**	**2**	**12**		**5**	**6**	**2**		**13**		**1:1.08:0.00**
**Nov**			**3**	**2**		**5**		**4**	**2**			**6**		**1:1.20:0.00**
**Dec**	**2**	**8**	**4**	**3**		**17**	**9**	**8**	**1**	**1**		**19**		**1:1.12:0.00**
**Total No**	**9**	**35**	**53**	**30**	**8**	**135**	**14**	**51**	**50**	**21**	**8**	**144**	**6**	**1:1.07:0.04**

*EAS* early active stage, *LAS* late active stage, *RS* ripe (Maturing) stage, *PSS* partially spent (spawning) stage, *SS* spent stage, *(H)* hermaphrodite, *M* male, *F* female

### Gametogenesis

**Fig. 4:**
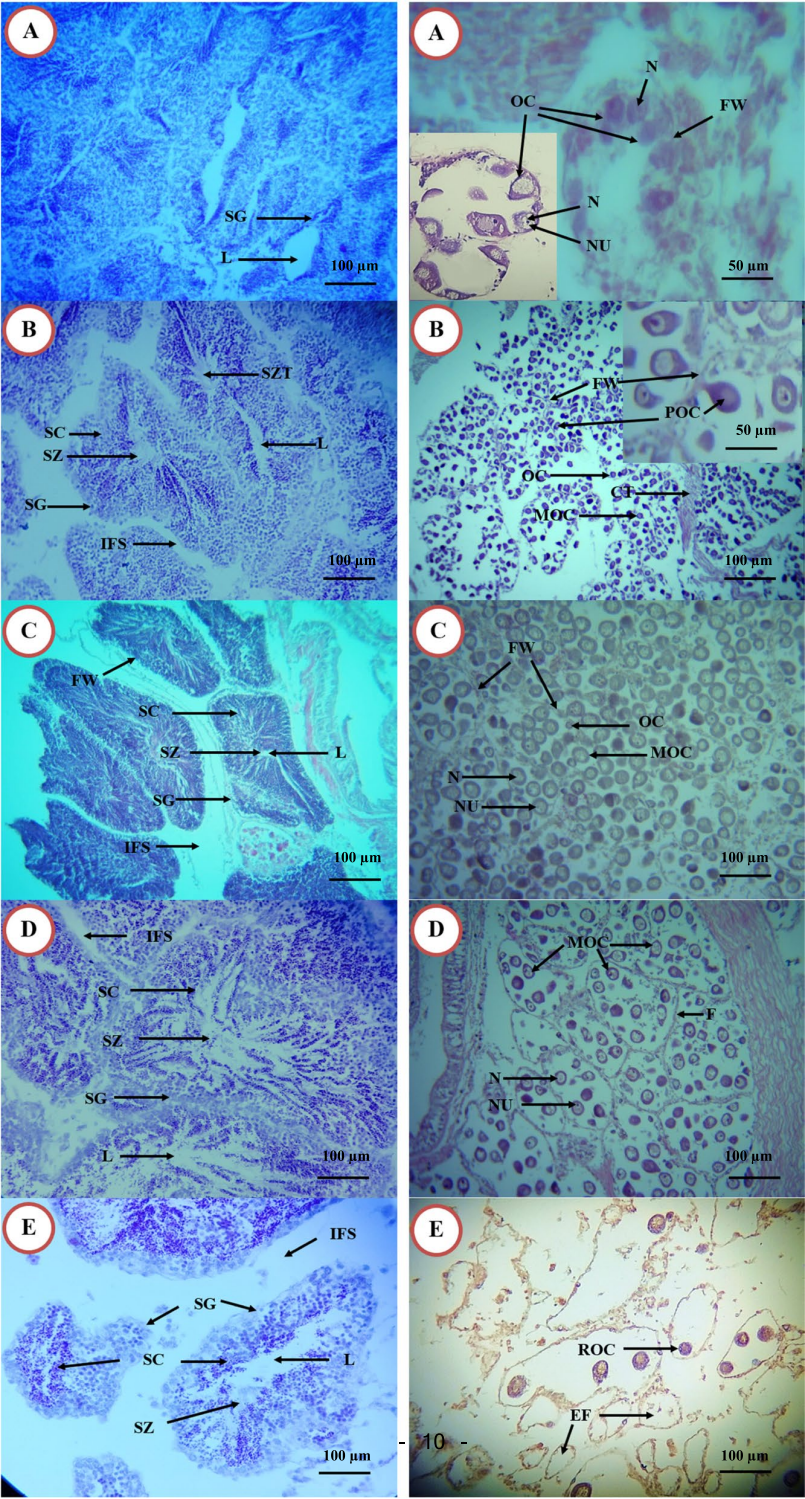
Photomicrograph of Gonadal development stages of *Paphia undulata* in both male and female with magnification 100 × except in A and enlarged portion of B in right panel (400X). Left panels: male; Right panels: female. A: early active stage; B: late active stage; C: ripe stage; D: partially spent stage; E: spent stage; CT: connective tissue; EF: empty follicle; F: follicle; FW: follicle wall; IFS: interfollicular space; L: lumen; MOC: mature oocyte; N: nucleus; NU; nucleolus; OC: mature oocyte; ROC: residual oocyte; SC: spermatocyte; SG: spermatogonia; SZ: spermatozoa; SZT: spermatozoa tail

Histological study of the gonads, can distinguish the sexes of *P. undulata*. It was found that gametogenesis of the calms in both males and females (Fig. 4) can be categorized into five stages as;

- Stage 1, Early active stage (EAS), in which sex can be distinguished and gametes proliferation began.

In females, oogonia characterized by a rounded shape with large nucleus surrounded by a thick layer of cytoplasm, the oogonia undergoes different stages of development, giving rise to oocytes, which are attached to the follicles’ wall, the oocytes are characterized by obvious chromatin nuclei, they are starting to fill the follicles, free oocytes absent in the lumen.

In males, rounded to expanded follicles filled with spermatogonia and spermatocytes were only present (Fig. 4A).

- Stage 2, Late active stage (LAS),

In females: Follicles are filled with mature free oocytes in the follicles’ lumens, appeared with peduncle, they have prominent nucleus and nucleolus. The amount of these free oocytes is less than half of the total number of follicles components; attached oocytes are abundant, in addition, there is small ration of oogonia.

In males: spermatogonia, spermatocytes, spermatids and spermatozoa are abundant in the follicles, Spermatids and spermatozoa can be observed but in small amounts by high magnification (Fig. 4B).

- Stage 3, Ripe (Maturing) Stage (RS),

In females, the follicles increase in size, occupy a large surface area and fuse with each other, many oocytes are free in the lumen of follicles, the shape of follicles is polygonal and their walls are thin epithelium layers.

In males, the follicles are mainly composed of large amounts matured spermatozoa with their flagellum pointing towards the center of the follicle, to form concentric bands or plugs; In very ripe specimens, spermatozoa bands are close to the follicle wall; the appearance of follicles are neat (Fig. 4C).

- Stage 4, Partially Spent (spawning) Stage (PSS),

In females: some follicles are empty due to the releasing of free oocytes; the follicle walls are broken (Fig. 4D).

In males: spermatozoa are clearly visible in a swirling shape and account for the greatest portion of cells in the follicle; there is empty space in some follicles due to the release of mature spermatozoa;

- Stage 5, Spent Stage (SS),

The follicles appear broken, scattered, and relatively empty,

In females: only residual oocytes found in the follicles, most of them are undergoing resorption; Many phagocytes are present (Fig. 4E).

In males, in advanced spent individuals, only residual spermatozoa are found in the lumen and undergoing resorption; there is a presence of phagocytes;

### Gonadal cycle

**Fig. 5:**
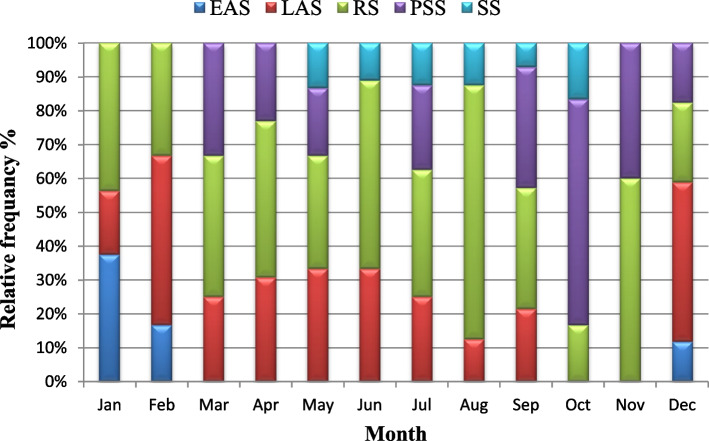
Monthly variation in the relative frequency of different spermatogenesis stages of male *Paphia undulata* from January to December 2020.EAS: early active stage; LAS: late active stage; RS: ripe (Maturing) stage; PSS: partially spent (spawning) stage; SS: spent stage

**Fig. 6:**
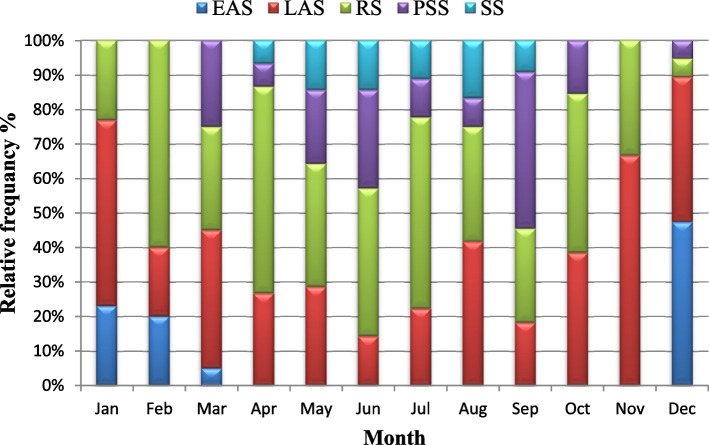
Monthly variation in the relative frequency of different oogenesis stages of female *Paphia undulata* from January to December 2020. EAS: early active stage; LAS: late active stage; RS: ripe (Maturing) stage; PSS: partially spent (spawning) stage; SS: spent stage

The study shows that *P. undulata* has a continuous breeding season as the co-occurrence of different reproductive stages in all the monthly samples of the population are observed (Table 1). The presence of mature gonads in almost all months and the infrequent occurrence of spent stages are consistent with this species having a prolonged reproductive cycle (Figs. 5 and 6). However, variation in the intensity of the reproductive activities results in peaks or periodicity in the reproductive phases.

The annual gonad development of male *P. undulata* was represented in Fig. 5. Male gonads in the early active development stage were observed from December to February although the late active development stage was observed throughout the year except in October and November. The ripe (maturing) stage was observed throughout the year without exception. Spawning activity began in March and continued into December except in June and August. The annual gonad development of female *P. undulate is* given in Fig. 6. Female gonads in the early active development stage were observed from December to March but the late active development stage and the ripe (maturing) stages were observed throughout the year. Spawning activity prolonged from March to December.

### Hermaphroditism

**Fig. 7:**
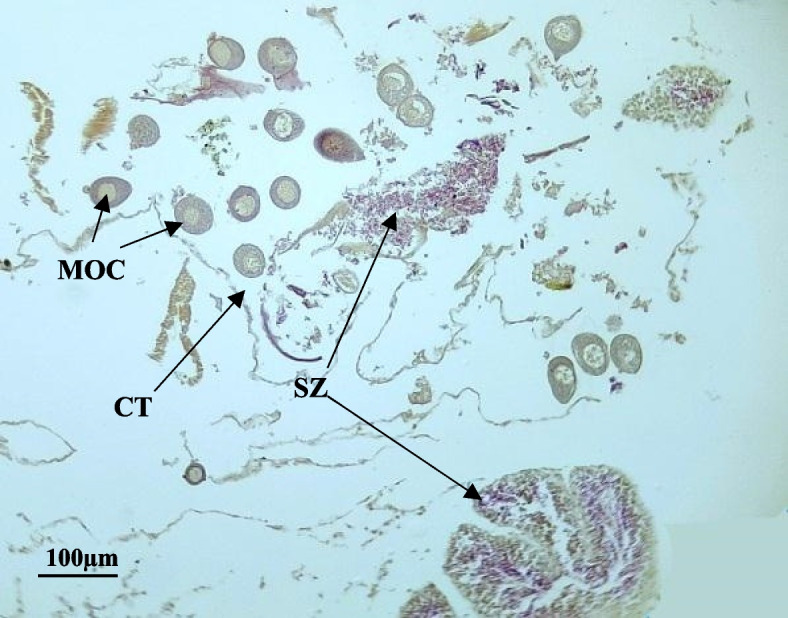
Photomicrograph of the hermaphrodite *Paphia undulata* shows spermatids and mature eggs, respectively. CT: connective tissue; SZ: spermatozoa; MOC: mature oocyte

In the current study, six specimens out of 286 specimens of *P undulata* were found to be hermaphrodite thus representing 2.1% of the studied sample. In all specimens, testicles and ovaries were found next to each other but separated, Fig. 7 showed that the hermaphrodite gonad contains testicles and ovaries in the partially spent stage.

## Discussion

The sex ratio (male:female) of the short-neck clam, *P. undulata*, was 1:1.07, which indicates that *P. undulata* is a dioecious organism. Some hermaphrodites were also encountered but in a low percentage. Males and females are equally represented in the population. This means that the population is still in equilibrium, wherein inbreeding and competition for mates does not occur [24, 25]. In general, the ratio of the sexes for dioecious bivalves is approximately 1:1 with the number of females often being slightly higher than the number of males [24, 26]. In some bivalves like *Lithophaga lithophaga* the ratio of males often being higher than the ratio of females (3.5:1) [27]. Previous studies on bivalve reproduction have found that the sex ratio was 1:1, including for *Gafrarium pectinatum* [28], *Gafrarium tumidum* [22], *Donax trunculus* [29], *Anadara granosa* [30], Blood cockles *Anadara inaequivalvis* [31], *Scrobicularia plana* [32], and Noah’s ark shell *Arca noae* [33].

Histological study of the gonads could distinguish sexes of *P. undulata*. It was found that *P. undulata* developed their gametes in different stages similar to other bivalves e.g. cockle, hard clam and manila clam etc. [25]. However, the duration of spawning was different, for example**,** most of the clams in temperate areas spawned in the spring and summer months as *Pinctada fucata martensii*, while the tropical clams spawned throughout the year [25].

*P. undulata* is a dioecious organism, with small numbers of hermaphrodites (2.1%). Nabuab et al. [24] found a small percentage of hermaphrodite (0.4%) at the same previous species, in the Central Philippines, but that species is still functionally dioecious. Kongasa and Drummond et al. [25, 34] interpreted the phenomenon of hermaphrodites in a dioecious mollusc as an unusual incidence. The unnatural condition of the environment may have caused that phenomenon. The sex change of clams has not been reported in short-necked clams *P. undulata* [21, 24].

*P. undulata* has a protracted or continuous breeding season as supported by the co-occurrence of different reproductive stages in all the population monthly samples. The presence of in almost monthly mature gonads and infrequent occurrence of spent stages is consistent with this species. However, variation in the intensity of the reproductive activities results in peaks or periodicity in the reproductive phases. Protracted breeding seasons with periodicity over an annual cycle are commonly exhibited by tropical bivalve species such as *Scapharca inequivalvis* [35], *Anodontia edentula* [36], and *Gari elongata* [37]. This is because seasonal patterns of gonad development result from responses to within-year changes in environmental factors [38, 39], although seasonal variation in the tropics may be minimal. The continuous gamete production in *P. undulata* may be a result of year-round food availability in the Suez Canal and Timsah Lake. *Venerupi saurea* and *Tapes decussata* have both been seen to produce gametes continuously and to spawn repeatedly in Timsah Lake due to the availability of food [40].

The onset size of sexual maturity varies both in the different species and same species under different ecological conditions [16]. The average size at the onset of sexual maturity of *P. undulata* in Timsah Lake was 2.5 cm and 2.1 cm for the females and males respectively. This value is greater than that of Sutthakorn and Tuaycharoen [41] who found that the smallest size at first maturity for the same species along the western coast of Thailand is 1.43 cm, while this is different from the Gulf of Thailand as in Trat province the size at first maturity is 4.25 cm. In addition, it is decreased to 3.2 cm as reported by Chatananthawej [42] in the same site and in Suratthani province it is 3.06 cm. Jindalikit [27] stated that the size at first mature of *P. undulata* in Mahachai Bay, Samut Sakhon province was 3.16 and 3.19 cm in both males and females, respectively while its size at first maturity found in Southern Negros Occidental, Central Philippines was 4.23 cm for males and 4.48 cm for females [43] and it was 4.26 cm for males and 4.48 cm for females [24]. The average size at sexual maturity in *P. undulata* was greater than 4.0 cm in Thailand [44]. However, additional studies are necessary to detect the relationship between an accurate age at the onset of sexual maturity and environmental factors in bivalves, especially temperature and latitude.

## Conclusion

In conclusion, the reproductive cycle, gametogenesis, and spawning season of *P. undulata* from Timsah Lake were done around one year. It was found that this species developed its gametes in different stages, and has a prolonged reproductive cycle. *P. undulata* is a dioecious organism with small numbers of hermaphrodites. The sex change of clams has not been reported. The resultant information recommended to support and develop large-scale artificial breeding.

## Data Availability

Data and Materials are available from Mostafa A. M. Mahmoud on reasonable request.
